# Supplementary figures and images for: Correlation of age and the diameter of the cervical nerve roots C5 and C6 during the first 2 years of life analyzed by high‐resolution ultrasound imaging
Source: Brain Behav. 2022 Jul 10;12(8):e2649. doi: 10.1002/brb3.2649 (PMC9392521; doi:10.1002/brb3.2649)

A

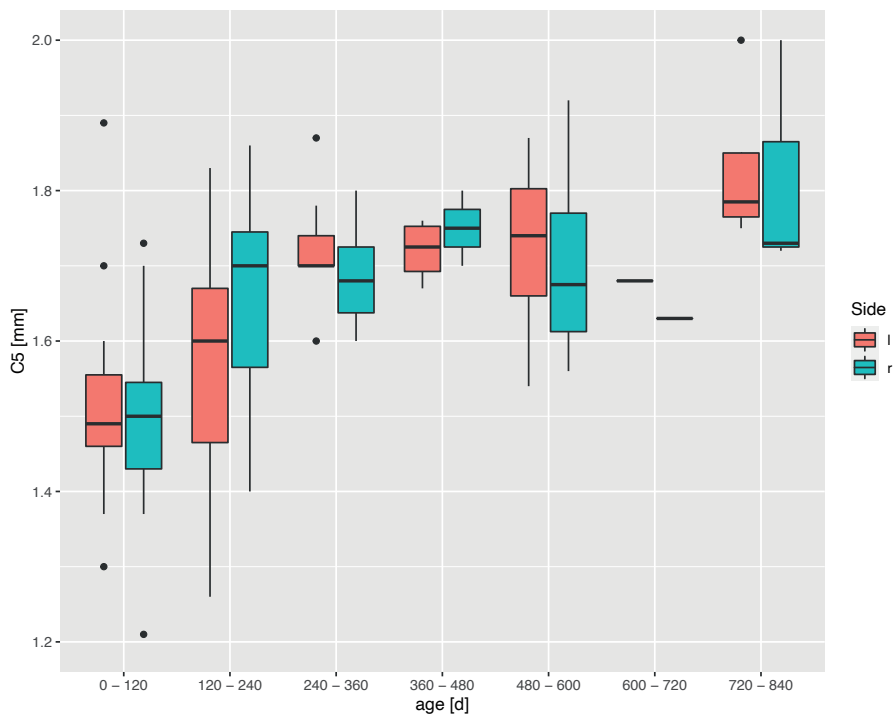

B

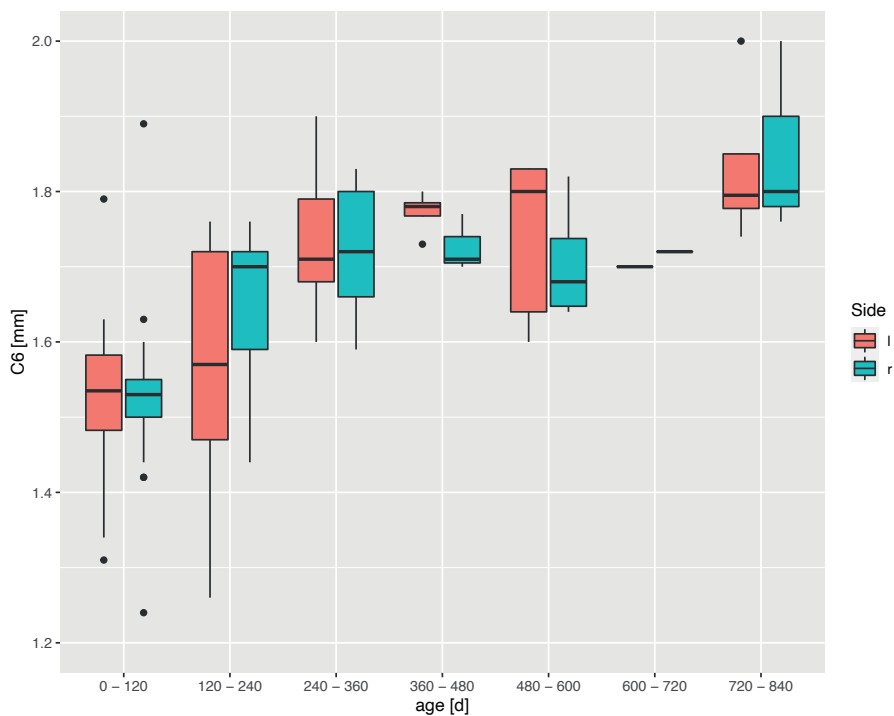

Supplementary Figure 01

Supplement: Supplementary file 1 — Supplementary Figure 1. Boxplots for the median diameter of the nerve roots C5 (Panel A) and C6 (Panel B) in relation to age. [file BRB3-12-e2649-s002.pdf]

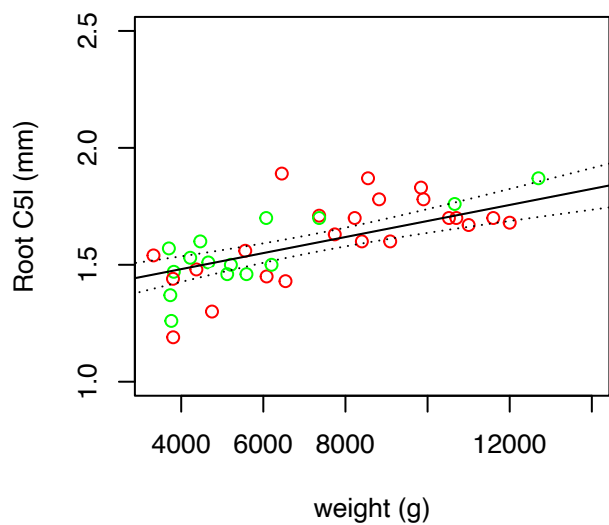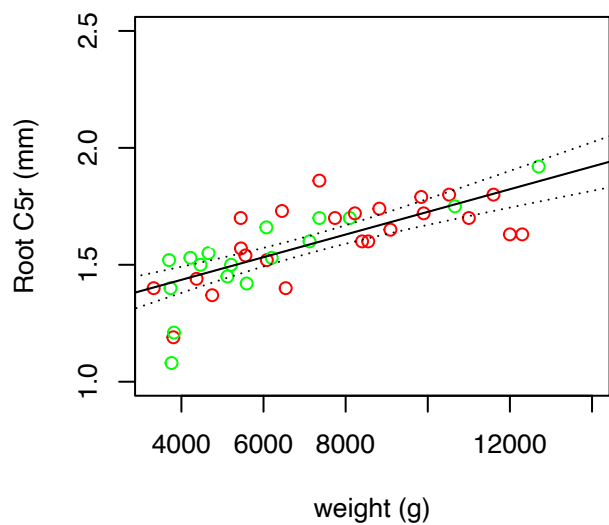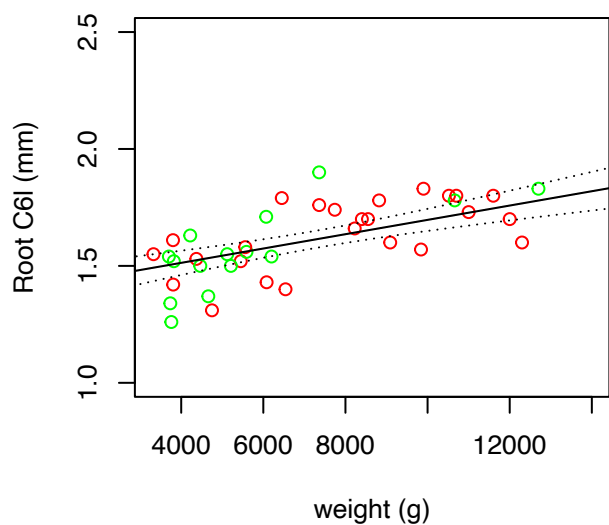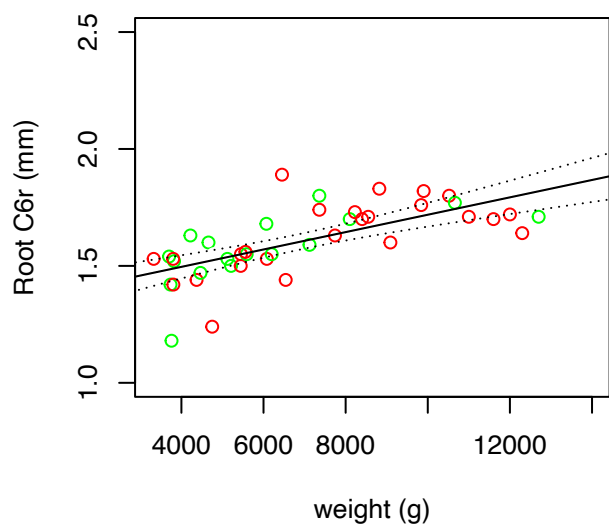

Supplemenray Figure 02

Supplement: Supplementary file 2 — Supplementary Figure 2. Diameter of the nerve roots C5 and C6 in relation to weight (green dots right side, red dots left side). The black line shows the linear regression curve with the 1.96 upper and lower of the standard error of the predicted means. [file BRB3-12-e2649-s001.pdf]
